# Detecting pediatric appendicular fractures using artificial intelligence

**DOI:** 10.1590/1806-9282.20240523

**Published:** 2024-08-30

**Authors:** Nezih Kavak, Rasime Pelin Kavak, Bülent Güngörer, Berna Turhan, Sümeyya Duran Kaymak, Evrim Duman, Serdar Çelik

**Affiliations:** 1 Etlik City Hospital, Department of Emergency – Ankara, Turkey.; 2 Etlik City Hospital, Department of Radiology – Ankara, Turkey.; 3 Etlik City Hospital, Department of Orthopedics and Traumatology – Ankara, Turkey.; 4 Ostim Technical University, Department of Management Information Systems – Ankara, Turkey.

**Keywords:** Artificial intelligence, Deep learning, Pediatrics. Radiography

## Abstract

**OBJECTIVE:**

The primary objective was to assess the diagnostic accuracy of a deep learning-based artificial intelligence model for the detection of acute appendicular fractures in pediatric patients presenting with a recent history of trauma to the emergency department. The secondary goal was to examine the effect of assistive support on the emergency doctor’s ability to detect fractures.

**METHODS:**

The dataset was 5,150 radiographs of which 850 showed fractures, while 4,300 radiographs did not show any fractures. The process utilized 4,532 (88%) radiographs, inclusive of both fractured and non-fractured radiographs, in the training phase. Subsequently, 412 (8%) radiographs were appraised during validation, and 206 (4%) were set apart for the testing phase. With and without artificial intelligence assistance, the emergency doctor reviewed another set of 2,000 radiographs (400 fractures and 600 non-fractures each) for labeling in the second test.

**RESULTS:**

The artificial intelligence model showed a mean average precision 50 of 89%, a specificity of 92%, a sensitivity of 90%, and an F1 score of 90%. The confusion matrix revealed that the model trained with artificial intelligence achieved accuracies of 93 and 95% in detecting fractures, respectively. Artificial intelligence assistance improved the reading sensitivity from 93.7% (without assistance) to 97.0% (with assistance) and the reading accuracy from 88% (without assistance) to 94.9% (with assistance).

**CONCLUSION:**

A deep learning-based artificial intelligence model has proven to be highly effective in detecting fractures in pediatric patients, enhancing the diagnostic capabilities of emergency doctors through assistive support.

## INTRODUCTION

Fractures constitute the most prevalent form of injury, with data indicating that from 40 to 60% of male children and 25 to 40% of female children will seek medical attention at an emergency department (ED) for a fracture^1^. These injuries are a significant contributor to long-term disability among the pediatric population.

Radiography stands as the primary and most frequently utilized imaging technique for fracture diagnosis. However, the failure or delay in diagnosing fractures in pediatric patients via radiograph is a common issue. This can be attributed to several challenges, including difficulty in ensuring appropriate positioning for children, the unavailability of comparative radiography due to limiting radiation exposure, and natural variability in the appearance of developing bones and growth plates in immature skeletons. These factors can lead to misinterpretation, where normal anatomical features may occasionally appear as injuries^2^.

Artificial intelligence (AI) is increasingly recognized for its potential to revolutionize medical diagnostics. An expanding corpus of research illustrates that AI software, particularly those based on deep learning methodologies, can achieve diagnostic accuracies in fracture detection from imaging studies that are on par with medical practitioners^3^. However, the field of pediatric fracture detection still lacks substantial research contributions^4^.

The primary objective was to assess the diagnostic accuracy of a deep learning-based AI model for the detection of acute appendicular fractures in pediatric patients presenting with a recent history of trauma to the ED. The secondary goal was to examine the effect of assistive support on the emergency doctor’s ability to detect fractures.

## METHODS

Following ethical committee approval, the study retrospectively analyzed 7,150 plain anteroposterior radiographs of patients aged 2–18 years (mean age 8.3 years) who presented to the Dışkapı Yıldırım Beyazıt Research and Training Hospital’s ED due to trauma between January 15, 2015, and December 30, 2020. The inclusion criterion was the availability of a radiograph of an appendicular part taken after a recent trauma, regardless of whether a fracture was present or not. Radiographs featuring implants, casts, or any other pathological lesions in the bones, as well as patients presenting fractures highly specific to child abuse (e.g., metaphyseal corner fractures), were excluded.

In the present study, a convolutional neural network (CNN) model known as You Only Look Once (YOLO)v8, which employs deep learning techniques, was applied to identify fractures within radiographs. The study involved the utilization of image enhancement, data balancing, preprocessing techniques, and radiograph training by using a YOLO-based framework. The YOLO architecture distinguishes itself by applying a neural network across the entire image to identify objects and encircle them with bounding boxes, instead of focusing on specific sections of the image. The radiographs were initially downloaded in the Digital Imaging and Communications in Medicine (DICOM) format, then converted into the Joint Photographic Experts Group (JPEG) format, and standardized to a resolution of 640×640 pixels.

In the study, bounding boxes to indicate detected fractures in radiographics were independently drawn by three radiologists (RPK, BT, and SDK) with experiences of 16, 10, and 9 years, using a specialized software devoid of AI assistance. The consensus criterion for a verified fracture involved an agreement among the experts’ bounding boxes, requiring an intersection over union ratio of more than 50%. To train the deep learning models, augmentation techniques were applied as part of the preprocessing strategies to enhance the training data pool.

**Figure 1:**
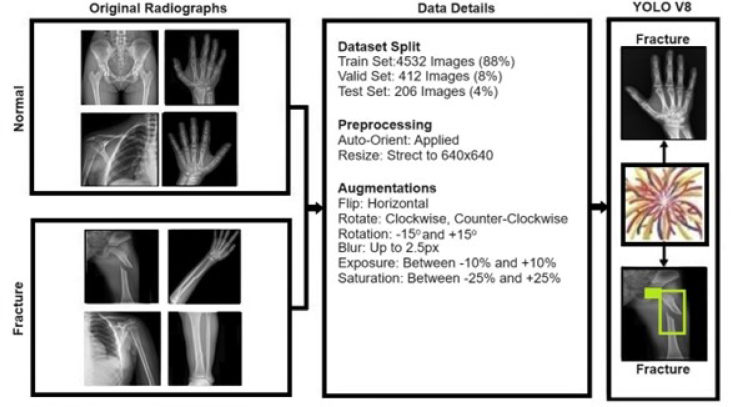
Workflow of data used in YOLOv8.

The dataset was 5,150 radiographs. Since deep learning algorithms require a large amount of labeled data to be trained, augmentation techniques, one of the preprocessing methodologies, have been used to enrich the training dataset (Figure 1).

An additional subset of 2,000 radiographs were reserved specifically for second testing and subsequent statistical evaluation. An emergency doctor (NK, 15 years of experience) conducted analyses on this dataset, performing assessments in two scenarios: without AI support (comprising 400 radiographs with fractures and 600 without) and with AI support (comprising 400 radiographs with fractures and 600 without), followed by an annotation process. For each radiograph, metrics of sensitivity and specificity were derived, facilitating a comparative analysis between the AI-assisted and non-assisted evaluations.

### Statistical method

The area beneath the receiver operating characteristic (ROC) curve was assessed using a dedicated web-based ROC analysis application, which utilizes a Python script. Sensitivity and specificity values were extracted using the optimal cutoff point determined by the area under the curve (AUC) analysis. The model’s performance metrics, including mean average precision (mAP), precision, recall, and the F1 score (which represents the harmonic mean between precision and recall), were derived using foundational parameters such as true positives (TP), true negatives (TN), false positives (FP), and false negatives (FN). Each image was evaluated through the finalized model for fracture detection, producing a probabilistic score on a scale from 0 to 1 for categorizing the image as fracture or no fracture.

## RESULTS

The design of the YOLOv8 model yielded significant performance metrics, including a mAP50 of 89%, specificity at 92%, sensitivity reaching 90%, and an F1 score of 90%. The analysis of the confusion matrix from testing data revealed that the YOLOv8-informed model attained accuracies of 93 and 95% in identifying fractures (Table 1). Furthermore, the integration of AI with expert evaluation from an emergency doctor enhanced the sensitivity of assisted readings to 97.0%, marking an improvement of 3.3% over the sensitivity of readings without AI assistance, which stood at 93.7%. Similarly, the accuracy of readings with AI support was elevated to 94.9%, surpassing the accuracy of unassisted readings by 6.9%, which was previously 88% (Table 2).

**Table 1:** YOLOv8 model performance comparison based on the training datasets.

You Only Look Once v8
	**Artificial intelligence**								
		**Total sets**	**True positive**	**True negative**	**False positive**	**False negative**	**Sensitivity (95%CI)**	**Specificity (95%CI)**	**Accuracy (95%CI)**
	All	5,150	3,712	921	352	163	95.8%	72.3%	90.0%
	Fracture	850	506	259	72	13	97.5%	78.2%	90.0%
	Not fracture	4,300	3,208	662	280	150	95.5%	70.3%	90.0%

**Table 2:** Emergency doctor and YOLOv8 model performance comparison based on the test datasets.

Emergency doctor
	**Without artificial intelligence**								
		**Total sets**	**True positive**	**True negative**	**False positive**	**False negative**	**Sensitivity (95%CI)**	**Specificity (95%CI)**	**Accuracy (95%CI)**
	All	1,000	655	230	71	44	93.7%	75.2%	88.0%
	Fracture	400	225	118	38	19	92.2%	75.6%	85.8%
	Not fracture	600	430	112	33	25	94.5%	77.2%	90.3%
	**With artificial intelligence**								
		**Total sets**	**True positive**	**True negative**	**False positive**	**False negative**	**Sensitivity (95%CI)**	**Specificity (95%CI)**	**Accuracy (95%CI)**
	All	1,000	688	261	30	21	97.0%	89.7%	94.9%
	Fracture	400	239	132	21	8	96.8%	86.3%	92.8%
	Not fracture	600	449	129	9	13	97.2%	93.5%	96.3%

## DISCUSSION

The ED acts as the primary point of contact for the initial assessment of fractures in pediatric patients arriving due to trauma. The frequent oversight of fractures in these settings is often a consequence of the demanding workloads, lengthy hours, and the medical staff’s insufficient training in analyzing radiographs. Such omissions can lead to postponements in administering the appropriate treatment, thereby escalating morbidity and the economic impact on the healthcare industry. In recent years, there has been an accelerated integration of AI across a diverse array of medical specialties. Nevertheless, the adoption of AI in pediatric radiology has been comparatively slow. This delay may be attributed to pediatric radiology’s relatively smaller role in healthcare, a greater diversity of case types, and generally lower case volumes, which hinder the collection of substantial datasets necessary for algorithm training^5^. This study revealed that the specified AI subset, leveraging deep learning, exhibited robust diagnostic capabilities in identifying appendicular fractures in pediatric patients. Moreover, it was observed that the diagnostic effectiveness of emergency doctors is notably enhanced when supplemented with AI software, surpassing the outcomes achieved by either AI or emergency doctors operating independently.

In the study by Duron et al.^6^, the AI system underwent training using 60,170 radiographs from 18 years or older trauma patients. A group comprising six radiologists and six emergency doctors was tasked with identifying and pinpointing fractures in 600 radiographs, half of which were aided by AI. The assistance from AI led to an 8.7% increase in the doctors’ sensitivity and a 4.1% enhancement in specificity. Additionally, it significantly reduced the incidence of FP by 41.9% among patients without fractures and decreased the average reading time by 15.0%. Notably, the independent performance of the AI system surpassed that of all manual readers, including expert radiologists in skeletal imaging, achieving an AUC of 0.94.

In their study, Hayashi et al.^7^ employed an AI program, BoneView™, to analyze radiographs from pediatric patients (aged 2–21 years), comprising 150 cases with fractures and 150 cases without fractures across various anatomical sites (hand/wrist, elbow/upper arm, shoulder/clavicle, foot/ankle, leg/knee). The findings demonstrated a patient-based sensitivity of 91.3%, a specificity of 90.0%, a fracture-based sensitivity of 92.5%, and an FP rate of 0.11 per patient among those without fractures. The AUC for all fractures on a per-patient basis was 0.93.

In a separate study by Dupuis et al.^8^, involving 5,865 radiographs from patients (aged 0–17 years), the diagnostic accuracy of the deep learning algorithm (Rayvolve) was evaluated, with the senior radiologist’s diagnosis serving as the gold standard. The algorithm exhibited a sensitivity of 95.7%, a specificity of 91.2%, and an overall accuracy of 92.6% in detecting fractures. Notably, the algorithm’s performance improved in patients older than 4 years. This discrepancy in performance was ascribed to the potential limitation that the training dataset might not have adequately represented the heterogeneity of the target demographic, particularly noting that young infants possess more cartilaginous structures, which could affect diagnostic outcomes.

In their research involving 480 patients and conducting 60 examinations per body region, Guermazi et al.^9^ assigned 24 readers to evaluate the entire validation dataset (n=480) both with and without AI assistance. The findings revealed that the sensitivity per patient increased by 10.4% with AI assistance, reaching 75.2%, compared to 64.8% without AI. Additionally, the specificity per patient was 95.6% with AI support, demonstrating non-inferiority to the 90.6% specificity observed without AI assistance, marking a significant difference of 15.0%. The incorporation of AI was also found to reduce the average examination reading time by 6.3 s. The research was structured with a predetermined fracture prevalence of 50% within the sample, thereby preventing the determination of positive or negative predictive values. Additionally, the authors acknowledged the likelihood of a carryover effect, a consequence of employing a 1-month minimum washout period in their research approach.

We attribute the observed variations in specificity and sensitivity across different studies to a wide spectrum of patient ages, injury mechanisms, and algorithms, along with the specific inclusion and exclusion criteria utilized.

Further, our research observed that AI, when used in conjunction with emergency doctors, enhanced the sensitivity of assisted readings by 3.3% and the accuracy of assisted readings by 6.9% compared to readings without AI assistance. The application of AI resulted in an AUC of 0.93 when used by an emergency doctor. This underscores the substantial support AI provides to emergency medical practice by bolstering the diagnostic proficiency of emergency doctors, enhancing their decision-making accuracy, and streamlining their clinical workflow.

The AI algorithm exhibited a 7.6% rate of FP, whereas the combination of an emergency doctor and AI assistance was associated with a 3% rate of FP. This occurrence can be ascribed to multiple factors, such as the magnitude of the study population, and should be considered a critical metric when assessing the algorithm’s comprehensive precision and sensitivity. As the deployment of AI systems in medical diagnostic processes becomes more prevalent, minimizing such rates of FP will be imperative to enhance the reliability and applicability of these algorithms^10^.

Our study was retrospective. Radiologists and emergency doctors involved in the AI algorithm evaluation assessed the radiographs without access to the patient’s clinical histories. Additionally, a limitation of the algorithm was its inability to comparatively evaluate lateral radiographs or a series of radiographs. Patients exhibiting fractures with high specificity for indicators of child abuse (e.g., metaphyseal corner fractures) were not included in our study; these fractures were not present in our dataset, and the AI algorithm was not trained to detect them.

## CONCLUSION

The YOLOv8 model demonstrates substantial efficacy in identifying fractures among pediatric patients, particularly when used to augment the diagnostic capabilities of emergency doctors.

## INFORMED CONSENT

Retrospective study.

## ETHICAL APPROVAL

This study was performed in line with the principles of the Declaration of Helsinki. The approval was granted by the Bilkent City Hospital Clinical Research Ethics Committee (Ankara, Turkey) (ID number: 2023/3479).
